# Supplementary material for: Medicinal cannabis plant extract (NTI164) modifies epigenetic, ribosomal, and immune pathways in paediatric acute-onset neuropsychiatric syndrome
Source: Neurotherapeutics. 2026 Jan 8;23(1):e00828. doi: 10.1016/j.neurot.2025.e00828 (PMC12976522; doi:10.1016/j.neurot.2025.e00828)
Supplement: Multimedia component 1 [file mmc1.pdf]

SUPPLEMENTARY MATERIAL

Table of Contents

Appendix 1. Inclusion and exclusion criteria for FENPANS1 clinical trial. 2

Supplementary Table 1. Clinical pathology parameters for safety monitoring. 2

Supplementary Table 2. Adverse events reported during the treatment phase (initial 12 weeks) of the FENPANS1 study. 2

Supplementary Table 3. Questionnaires used in the FENPANS1 study. 3

Supplementary Table 4. Detailed statistics of clinical surveys. 3

Supplementary Figure 1. Clinical survey subdomain scores of PANS children at baseline and following 12 weeks of NTI164 administration. 4

Supplementary Figure 2. Plasma cytokine expression in PANS-pre and PANS-post compared to healthy controls. 6

Supplementary Figure 3. Exploratory bulk RNA sequencing and removal of unwanted variation. 6

Supplementary Figure 4. Single-cell RNA sequencing. 7

Supplementary Figure 5. Proteomics analyses. 8

Supplementary Figure 6. Phosphoproteomics analyses. 9

Supplementary Figure 7. Methylation analyses. 11

Appendix 1. Inclusion and exclusion criteria for FENPANS1 clinical trial.

Inclusion criteria:

- <18 years of age at time of enrolment
- Fulfill PANS criteria (Chang *et al.*, 2015):
    - Acute onset of OCD or severely restricted food intake
    - Concurrent presentation of additional neuropsychiatric symptoms from at least 2 of the following 7 categories: anxiety, emotional lability/depression, irritability, aggression or severely oppositional behaviours, behavioural regression, deterioration in school performance, sensory or motor abnormalities (e.g. tics), somatic symptoms (e.g. sleep disturbances, enuresis or increase in urine frequency)
    - Symptoms not better explained by a known neurologic or medical disorder (e.g. Sydenham's chorea)
- RCADS-P score of >65 (a scale of anxiety, social phobia, panic disorder, OCD, and low mood, and a score of >65 infers moderate-significant impairment)
- Other patient medications (e.g. anti-psychotics) must be stable for at least 12 weeks prior to trial participation

Exclusion criteria:

- Infection and/or antibiotic use in the 2 weeks prior to trial participation (i.e. baseline blood tests and commencement of NTI164)
- Recent changes to other patient medication (e.g. addition or escalation of anxiolytics, anti-depressants etc; medication must be stable for at least 12 weeks prior to trial participation)
- Ongoing immunomodulating or immunosuppressive treatment use in the previous 12 weeks, including steroids, IVIg, antibiotics, low-dose naltrexone, mycophenolate, Rituximab etc.
- Currently using or has used recreational or medicinal cannabis or cannabinoid-based medications (e.g. Sativex®, Epidiolex®) in the previous 12 weeks and/or is unwilling and/or unable to abstain for the duration of the trial
- Underlying renal impairment, cardiovascular issues (e.g. arrhythmia), current or previous thrombosis
- Impaired hepatic function, defined as serum alanine aminotransferase (ALT) or aspartate aminotransferase (AST) > 2 x upper limit of normal (ULN) or total bilirubin (TBL) > 2 x ULN; this criterion can only be confirmed once baseline laboratory values are available (i.e. post-enrolment) and participants who fail this criterion will not proceed in the study
- Other diagnosed neurological condition likely to be contributing to OCD/neuropsychiatric symptoms (e.g. Huntington's disease)

Supplementary Table 1. Clinical pathology parameters for safety monitoring.

**Haematology**	Haematocrit, haemoglobin, mean cell haemoglobin (MCH), mean cell volume (MCV), mean corpuscular haemoglobin concentration (MCHC), platelets, mean platelet volume (MPV), red blood cell (RBC) count, RBC distribution width, white cell count, basophils, eosinophils, lymphocytes, monocytes, neutrophils
**Clinical chemistry**	Albumin, alkaline phosphatase (ALP), alanine transaminase (ALT), anion gap, aspartate transferase (AST), bicarbonate, bilirubin (total, direct & indirect), calcium (adjusted), calcium, chloride, creatinine, estimated glomerular filtration rate (eGFR), gamma-glutamyl transferase (GGT), globulin, lactate dehydrogenase (LD), phosphorus, potassium, protein, sodium, urea, uric acid
**Coagulation**	International normalised ratio (INR), prothrombin time, activated partial thromboplastin time (aPTT)

Supplementary Table 2. Adverse events reported during the treatment phase (initial 12 weeks) of the FENPANS1 study.

Reported event	Mild	Severe	Total
Lethargy	1 (7%)	-	1 (7%)
Changes in sleep behaviour	2 (13%)	-	2 (13%)
Nausea	1 (7%)	-	1 (7%)

Diarrhoea	-	1 (7%)	1 (7%)

Supplementary Table 3. Questionnaires used in the FENPANS1 study.

Questionnaire	Outcomes measured
Clinical Global Impression-Severity (CGI-S)<br>Co-primary outcome<br>*Clinician-rated*	7-point scale rating clinician's impression of disease severity at a given time-point (1 = not at all ill, 7 = among the most extremely ill).
Clinical Global Impression-Improvement<br>Co-primary outcome<br>*Clinician-rated*	7-point scale rating clinician's impression of symptom change from baseline following an intervention (4 = no change, <4 = improvement, >4 = worsening).
Revised Children's Anxiety and Depression Scale-Parent (RCADS-P)<br>Co-primary outcome<br>*Parent-rated*	A 47-item questionnaire with subscales including separation anxiety disorder, social phobia, generalised anxiety disorder, panic disorder, obsessive-compulsive disorder, and low mood/major depressive disorder.
Children's Yale-Brown Obsessive Compulsive Scale-2nd Edition (CY-BOCS-II)<br>Co-secondary outcome<br>*Clinician-rated*	Gold-standard 19-item tool to rate severity of obsessive and compulsive symptoms in children and adolescents, 6-17 years of age.
Yale Global Tic Severity Scale (YGTSS)<br>Co-secondary outcome<br>*Clinician-rated*	Gold-standard for assessing tics in both children and adults. Evaluates the number, frequency, intensity, complexity, and interference of motor and phonic tics over the previous week.
Conner's rating scale – short version<br>Co-secondary outcome<br>*Parent-rated*	Assessment of symptoms and impairment associated with attention-deficit hyperactivity disorder (ADHD) in children and adolescents, 6-18 years of age. Assesses behavioural, social, and academic issues.
EQ-5D-Y<br>Co-secondary outcome<br>*Parent-rated*	Assesses function in five dimensions: mobility, looking after oneself, doing usual activities, having pain or discomfort, and feeling worried, sad, or unhappy.<br>Also assesses quality of life using a 0-100 visual analogue scale (0 = the worst health you can imagine, 100 = the best health you can imagine).

Supplementary Table 4. Detailed statistics of clinical surveys.

Survey		Mean		Median		Range		P-value (PANS-post vs pre)
		Baseline	Week 12	Baseline	Week 12	Baseline	Week 12	
CGI-S		4·8	3·3	5	3	4-6	2-5	<0·0001
CGI-I		4	2·8	4	3	4	2-4	<0·0001
RCADS-P	Total score	101·9	78·7	99	76	90-121	56-117	<0·0001
	Social phobia	19·7	11·8	21	11	5-26	3-23	<0·0001
	Panic disorder	11·4	6·1	10	5	7-22	2-15	0·018
	Major depression	18·9	12·9	19	13	12-26	4-26	0·0098
	Separation anxiety	15·2	8	16	6	6-21	1-16	<0·0001
	Generalised anxiety	13·6	7·5	14	6	8-17	3-18	<0·0001
	Obsessive-compulsive	11	6	11	7	4-18	1-12	0·001

CYBOCS-II	Total score	33·1	27·3	32	28	24-48	10-39	0·0023
	Obsessions	17·4	13·6	17·5	12·5	10-25	8-22	0·0012
	Compulsions	16	13·6	18	13	0-25	0-24	0·0994
YGTSS	Total score	44·1	14·8	49	13	15-75	0-34	<0·0001
	Motor tics	11·2	8·8	11	8	5-18	0-18	0·0584
	Vocal tics	4·3	4·2	0	4·5	0-23	0-10	0·9686
	Total tics	15·5	12·2	13·5	8	5-41	0-28	0·2479
Conner's		21·6	18	24	17	3-28	2-30	0·0216
EQ-5D-Y		9·9	8·8	10	9	7-13	5-12	0·0061

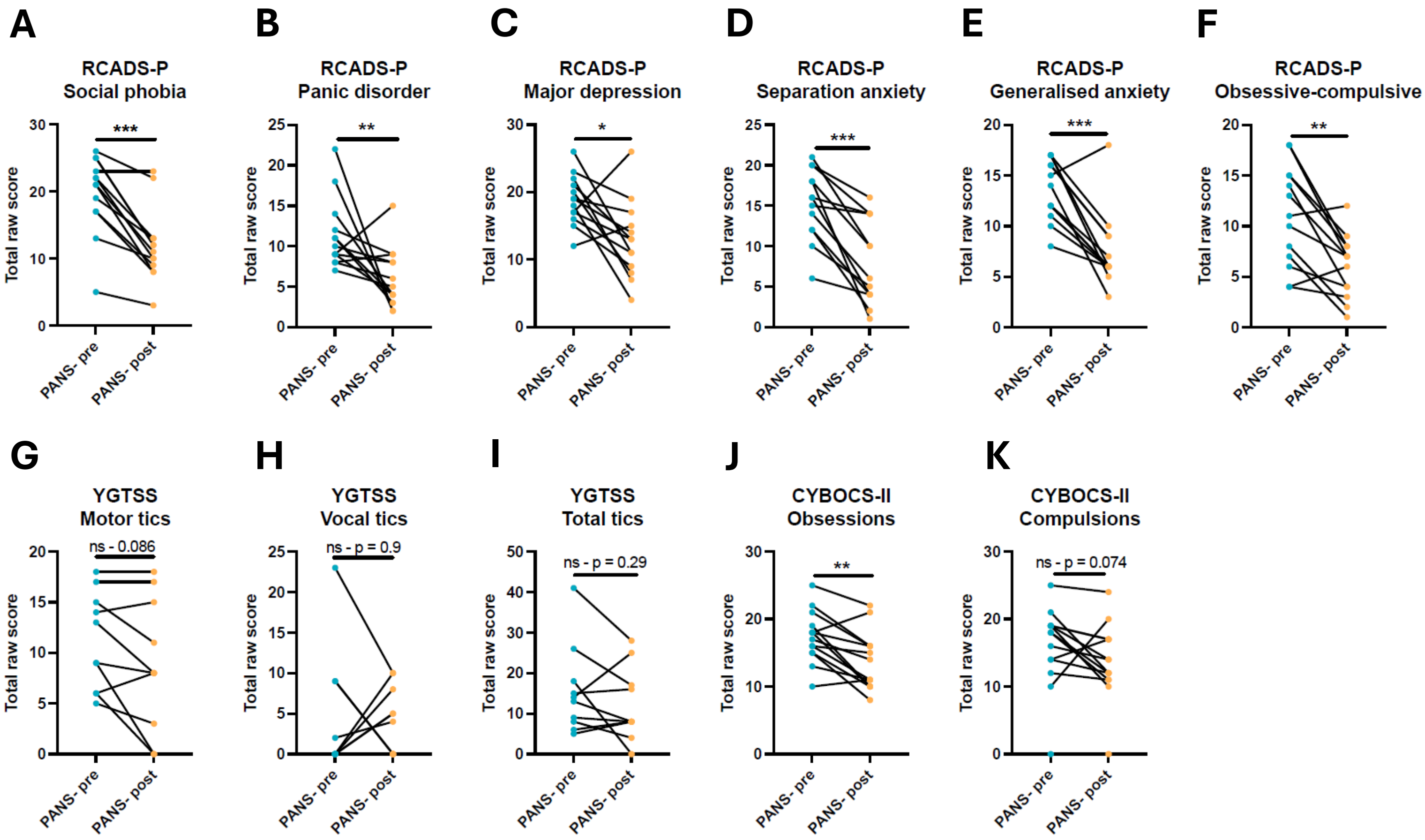

Supplementary Figure 1. Clinical survey subdomain scores of PANS children at baseline and following 12 weeks of NTI164 administration. Specific subdomains for gold-standard behavioural assessment scales were significantly improved following 12 weeks of NTI164 administration.

(a-f) RCADS-P subdomains: social phobia, panic disorder, major depression, separation anxiety, generalised anxiety, and obsessive-compulsive symptoms, respectively.

(g-i) YGTSS subdomains: motor tics, vocal tics, total tics, respectively.

(j-k) CYBOCS-II subdomains: obsessions, compulsions, respectively.

Wilcoxon matched-pairs signed rank test, n = 14, *P < 0·05, **P < 0·01, ****P < 0·0001.

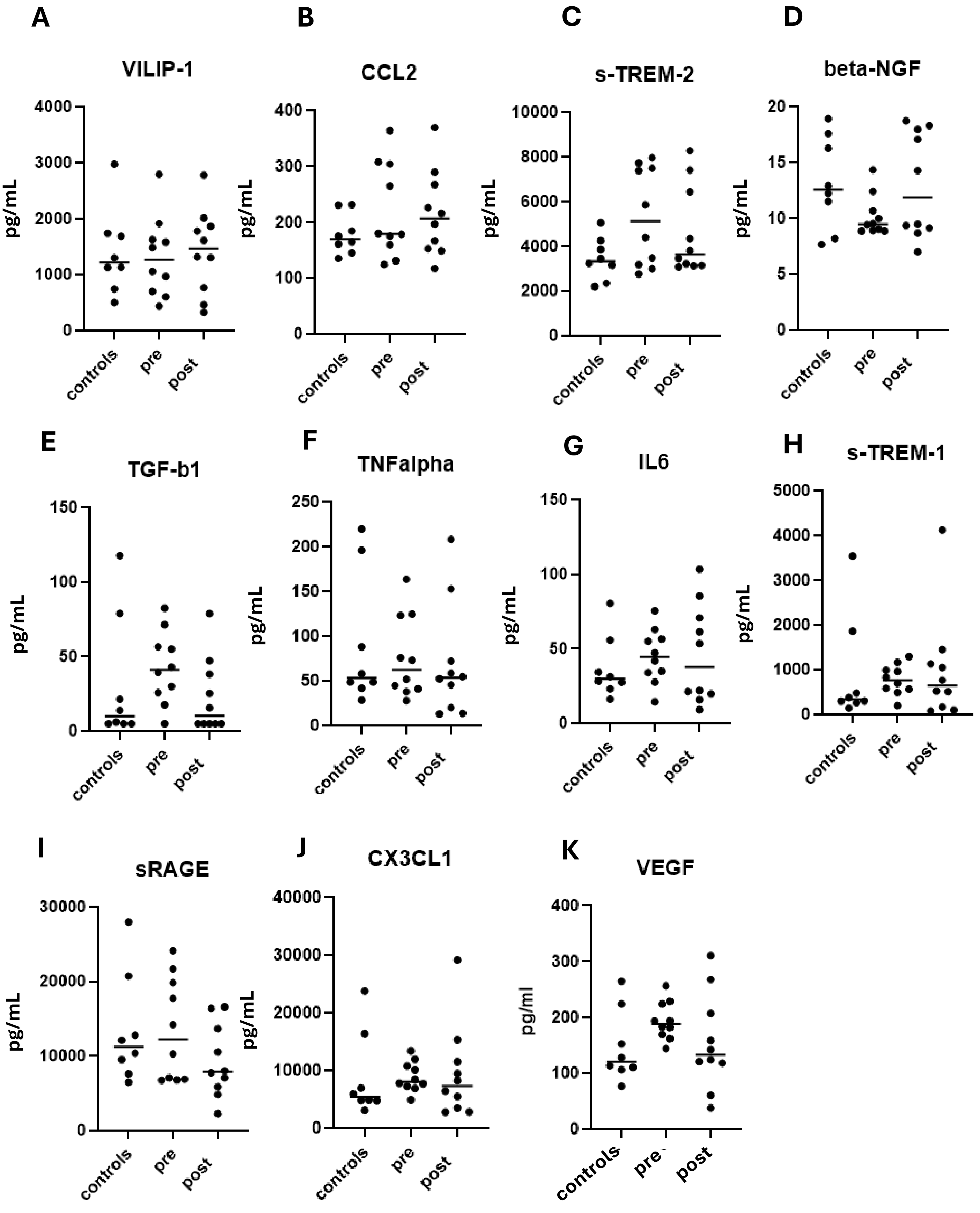
A
VILIP-1
pg/mL
4000
3000
2000
1000
0
controls
pre
post
B
CCL2
pg/mL
400
300
200
100
0
controls
pre
post
C
s-TREM-2
pg/mL
10000
8000
6000
4000
2000
0
controls
pre
post
D
beta-NGF
pg/mL
20
15
10
5
0
controls
pre
post
E
TGF-b1
pg/mL
150
100
50
0
controls
pre
post
F
TNFalpha
pg/mL
250
200
150
100
50
0
controls
pre
post
G
IL6
pg/mL
150
100
50
0
controls
pre
post
H
s-TREM-1
pg/mL
5000
4000
3000
2000
1000
0
controls
pre
post
I
sRAGE
pg/mL
30000
20000
10000
0
controls
pre
post
J
CX3CL1
pg/mL
40000
30000
20000
10000
0
controls
pre
post
K
VEGF
pg/ml
400
300
200
100
0
controls
pre
post

Supplementary Figure 2. Plasma cytokine expression in PANS-pre and PANS-post compared to healthy controls.
(a-k): Plasma cytokine expression levels of healthy control children, PANS-pre, and PANS-post: visinin-like protein 1 (VILIP-1), C-C motif ligand 2 (CCL2), soluble form of triggering receptor expressed on myeloid cells 2 (s-TREM-2), beta nerve growth factor (β-NGF), transforming growth factor beta (TGF-β1), tumour necrosis factor alpha (TNF-α), interleukin 6 (IL-6), s-TREM-1, soluble receptor for advanced glycation end products (sRAGE), chemokine (C-X3-C motif) ligand 1 (CX3CL1), and vascular endothelial growth factor (VEGF).

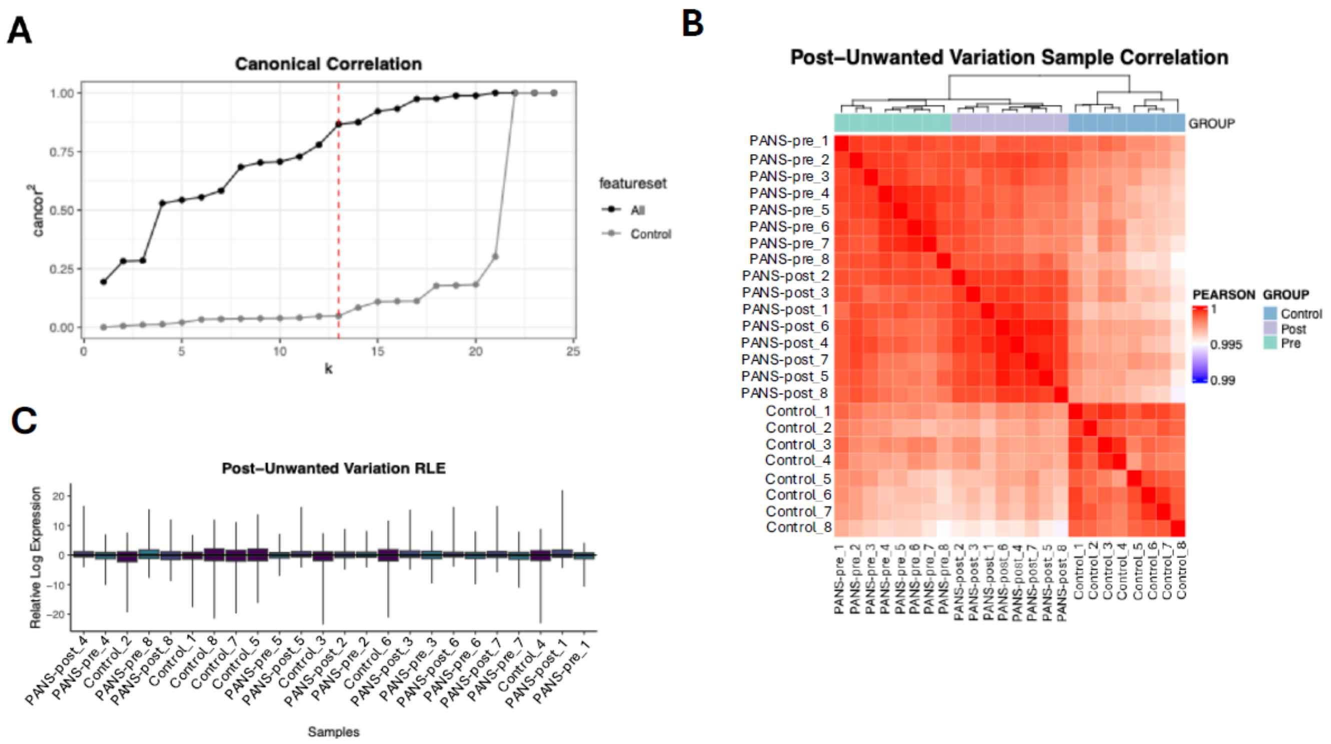

Supplementary Figure 3. Exploratory bulk RNA sequencing and removal of unwanted variation.
(a) In bulk RNA sequencing, k = 13 (factors of unwanted variation) were used to remove genes with minimal differential expression compared to negative control genes.
(b) Heatmap depicting high pearson correlation and clustering of samples within groups following removal of unwanted variation.
(c) Box and whisker relative log expression plot of samples after normalisation.

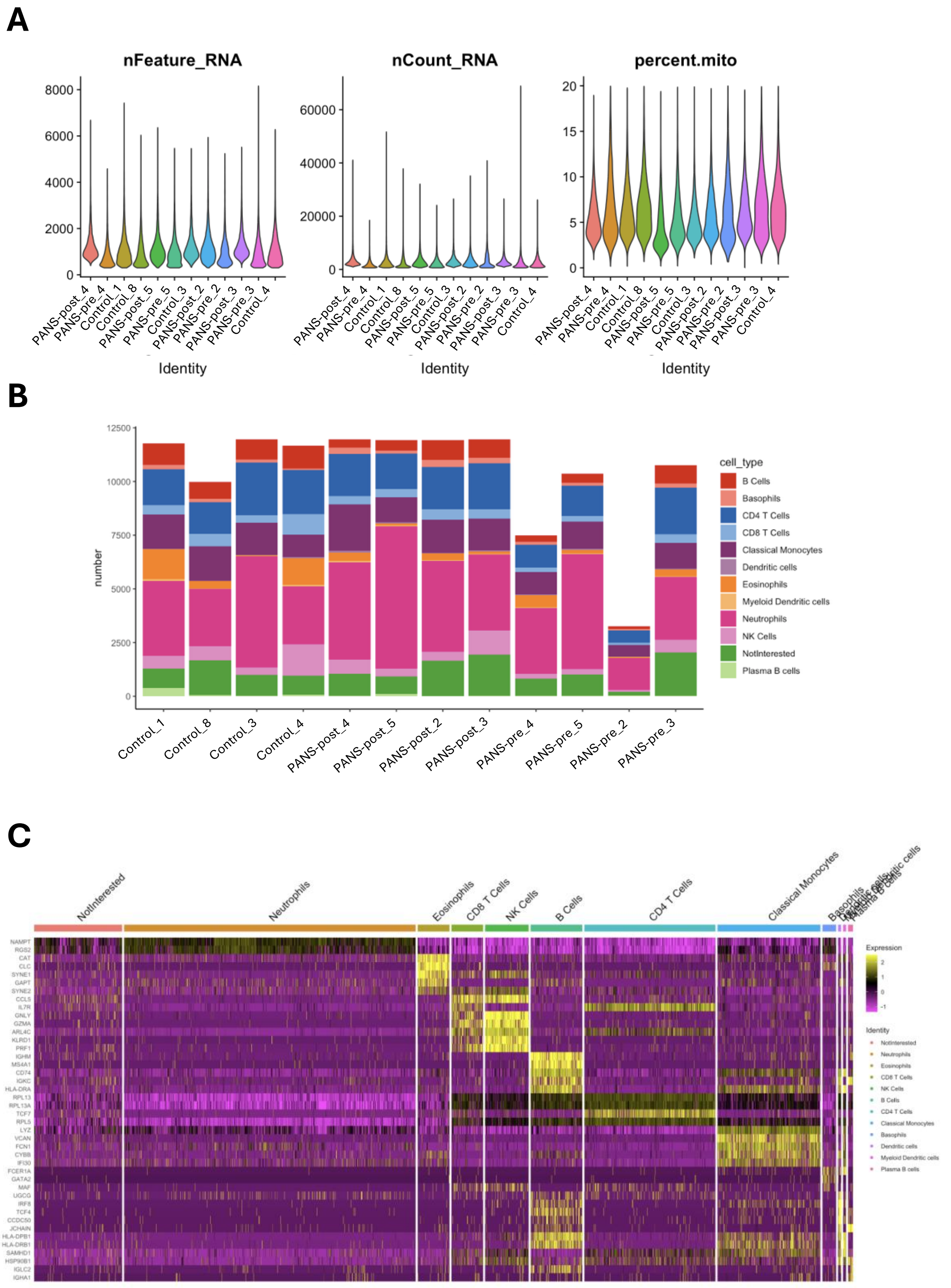

Supplementary Figure 4. Single-cell RNA sequencing.
(a) Number of features, counts, and percentage of mitochondrial genes per sample.
(b) Proportion of individual cell types per sample.
(c) Heat map of top differentially expressed genes based on cell type: genes are the top 5 markers of differential analysis between cell types (FDR <0·05) ranked by the difference in proportion of cells expressing the respective gene.

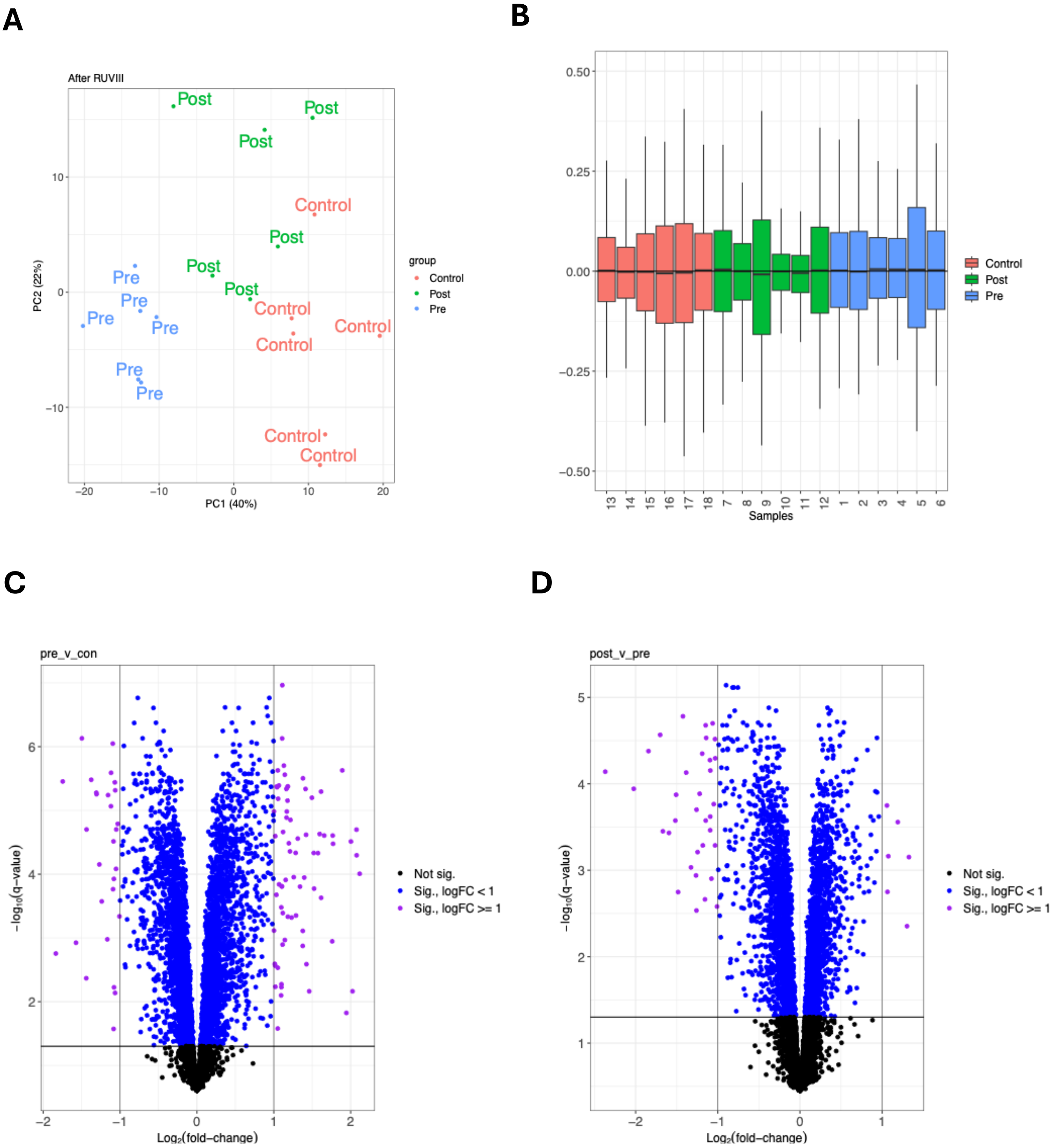

Supplementary Figure 5. Proteomics analyses.
(a) Principal component analysis (PCA) performed on proteomics shows clear discrimination between healthy controls, PANS-pre and PANS-post. The x-axis represents Principal Component 1 (PC1), while the y-axis represents Principal Component 2 (PC2).
(b) Box and whiskers relative log expression (y-axis) plot of samples after normalisation.
(c) Volcano plot of differentially expressed proteins (FDR <0·05) in the PANS-pre vs control comparison.
(d) Volcano plot of differentially expressed proteins (FDR <0·05) in the PANS-post vs pre comparison.

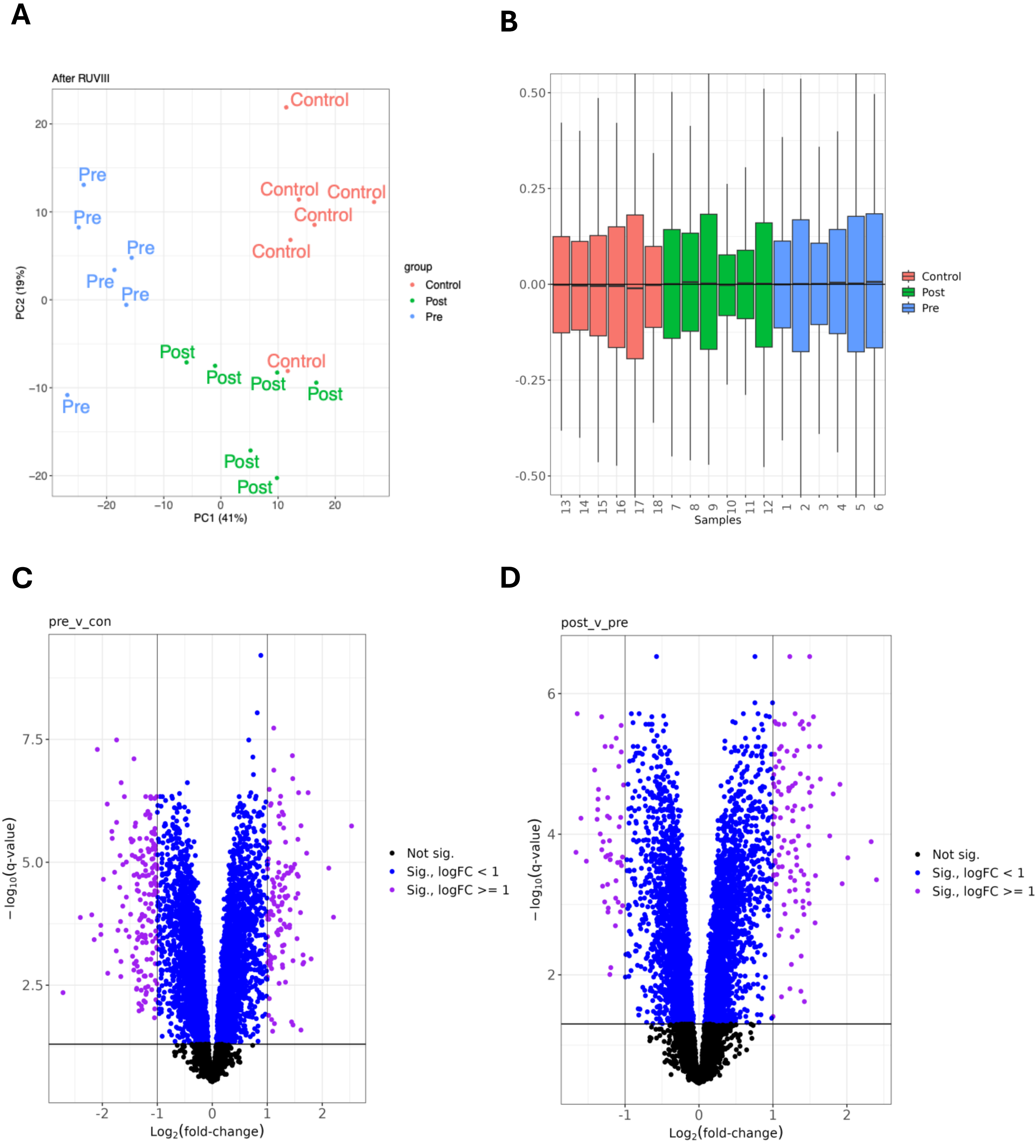

Supplementary Figure 6. Phosphoproteomics analyses.
(a) Principal component analysis (PCA) performed on phosphoproteomics shows clear discrimination between healthy controls, PANS-pre and PANS-post. The x-axis represents Principal Component 1 (PC1), while the y-axis represents Principal Component 2 (PC2).
(b) Box and whiskers relative log expression plot (y-axis) of samples after normalisation.
(c) Volcano plot of differentially expressed phosphopeptides (FDR <0·05) in the PANS-pre vs control comparison.
(d) Volcano plot of differentially expressed phosphopeptides (FDR <0·05) in the PANS-post vs pre comparison.

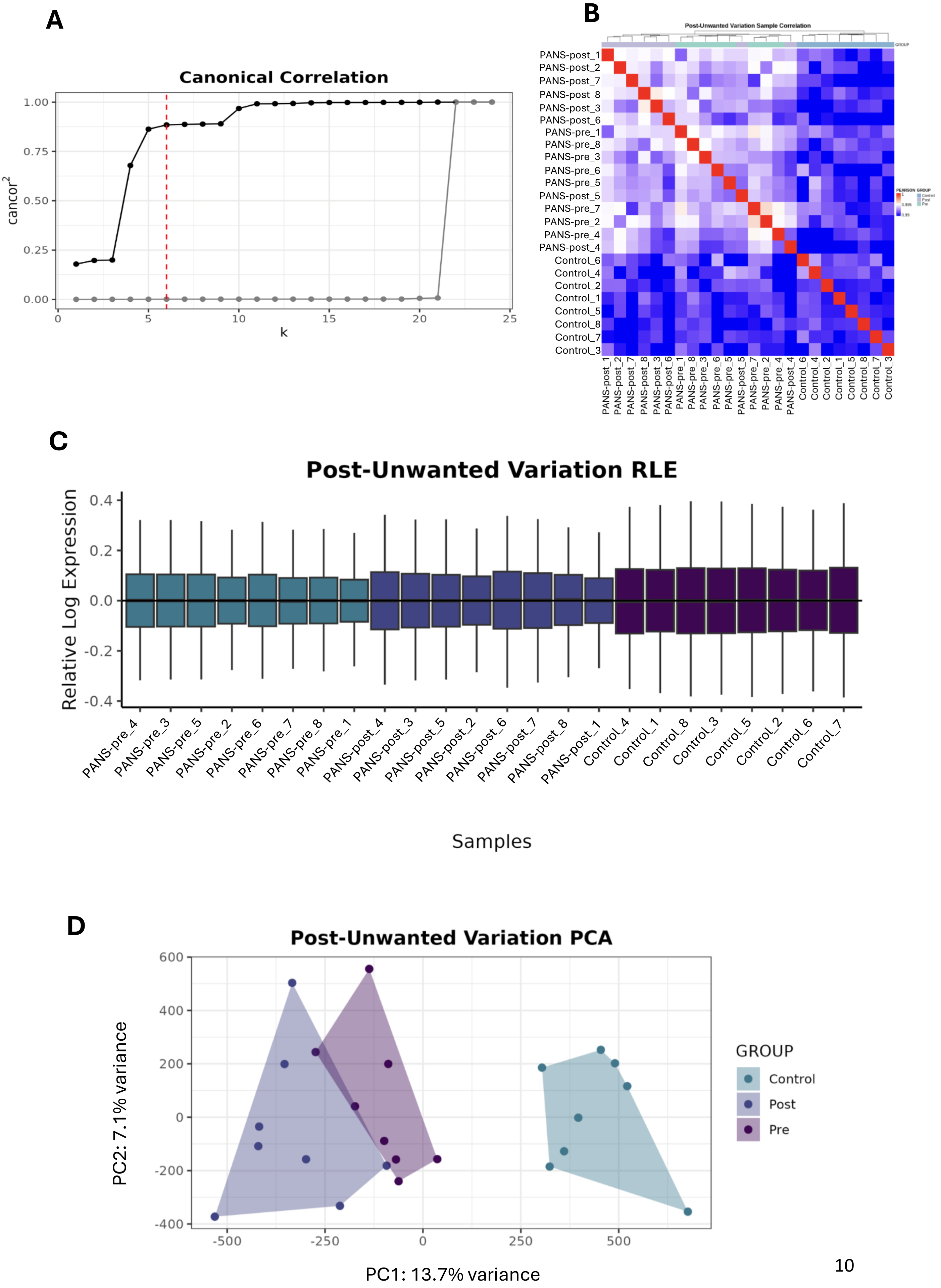
A
Canonical Correlation
cancor^2
k
1.00
0.75
0.50
0.25
0.00
0
5
10
15
20
25
B
Post-Unwanted Variation Sample Correlation
PANS-post_1
PANS-post_2
PANS-post_7
PANS-post_8
PANS-post_3
PANS-post_6
PANS-pre_1
PANS-pre_8
PANS-pre_3
PANS-pre_6
PANS-pre_5
PANS-post_5
PANS-pre_7
PANS-pre_2
PANS-pre_4
PANS-post_4
Control_6
Control_4
Control_2
Control_1
Control_5
Control_8
Control_7
Control_3
C
Post-Unwanted Variation RLE
Relative Log Expression
0.4
0.2
0.0
-0.2
-0.4
PANS-pre_4
PANS-pre_3
PANS-pre_5
PANS-pre_2
PANS-pre_6
PANS-pre_7
PANS-pre_8
PANS-pre_1
PANS-post_4
PANS-post_3
PANS-post_5
PANS-post_2
PANS-post_6
PANS-post_7
PANS-post_8
PANS-post_1
Control_4
Control_1
Control_8
Control_3
Control_5
Control_2
Control_6
Control_7
Samples
D
Post-Unwanted Variation PCA
PC2: 7.1% variance
PC1: 13.7% variance
600
400
200
0
-200
-400
-500
-250
0
250
500
GROUP
Control
Post
Pre

Supplementary Figure 7. Methylation analyses.
(a) For methylation data, k = 6 (factors of unwanted variation) were used to remove methylated positions with minimal differential expression compared to negative control methylated positions.
(b) Heatmap depicting high pearson correlation and clustering of samples within groups following removal of unwanted variation.
(c) Box and whisker relative log expression plot of samples after normalisation.
(d) Principal component analysis (PCA) performed on DNA methylation data shows clear discrimination between healthy controls, PANS-pre and PANS-post. The x-axis represents Principal Component 1 (PC1), while the y-axis represents Principal Component 2 (PC2).
